# Endoplasmic reticulum stress in Hashimoto’s thyroiditis: a candidate amplification node linking thyroid-specific vulnerability and immune dysregulation

**DOI:** 10.3389/fimmu.2026.1909257

**Published:** 2026-08-17

**Authors:** Xinying Si, Zhixun Guo, Gena Jiao, Xiaomei Zhang, Yaxue Ge, Jiahui Qian, Xinyi Li, Wenqian Geng, Simiao Yao, Zhiguo Ding, Yanke Wu

**Affiliations:** 1 Dongzhimen Hospital, Beijing University of Chinese Medicine, Beijing, China; 2 Sunsimiao Hospital, Beijing University of Chinese Medicine, Tongchuan, Shaanxi, China; 3 Beijing Health Vocational College, Beijing, China; 4 Beijing Tsinghua Changgung Hospital, School of Clinical Medicine, Tsinghua University, Beijing, China

**Keywords:** autoimmune thyroiditis, endoplasmic reticulum stress, Hashimoto’s thyroiditis, IRE1α, unfolded protein response

## Abstract

Hashimoto’s thyroiditis (HT) is the most prevalent autoimmune thyroid disease, characterized by lymphocytic infiltration and destruction of thyroid follicular architecture. Prior research has predominantly focused on aberrant adaptive immune activation, whereas the contribution of the thyroid follicular cell itself to this process remains incompletely elucidated. This narrative review synthesizes evidence implicating endoplasmic reticulum stress (ERS) in HT pathogenesis, stratifying the evidence by its proximity to HT pathology into three tiers: direct evidence, thyroid-cell evidence, and extrapolated evidence. Thyroid follicular cells continuously produce large quantities of structurally complex thyroglobulin, placing the ER under near-saturated folding load even under physiological conditions, thereby conferring an organ-specific vulnerability. Multiple HT-associated factors can activate the unfolded protein response (UPR), mediating thyrocyte apoptosis, suppression of functional genes, and loss of epithelial phenotype in thyroid cell models; individual UPR branches may also regulate the differentiation and function of infiltrating immune cells. Integrating the above evidence, this review proposes a testable positive-feedback perspective in which thyrocyte ERS injury and immune effector amplification may mutually reinforce one another. This perspective comprises six steps, and for each step, the strength of supporting evidence is assessed. Among these, only thyrocyte ERS activation and the macrophage IRE1α pathway have received preliminary direct evidence in HT or experimental autoimmune thyroiditis (EAT); support for the remaining steps derives primarily from thyroid cell lines or non-thyroid experimental systems.

## 1 Introduction

Hashimoto’s thyroiditis (HT) is the most prevalent autoimmune thyroid disease, pathologically characterized by lymphocytic infiltration, destruction of thyroid follicular architecture, and persistently positive thyroid-specific autoantibodies (TPOAb, TgAb). It represents the leading cause of hypothyroidism in iodine-sufficient regions (1). A systematic review and meta-analysis encompassing 48 studies and over 22 million individuals reported a global adult HT prevalence of approximately 7.5% (95% CI 5.7%–9.6%), with female risk approximately fourfold that of males (2). Moreover, HT is associated with increased cardiovascular event risk (3) and the development of papillary thyroid carcinoma (4); its disease burden has thus become a significant public health concern in endocrinology. The pathogenesis of HT arises from the interplay of genetic susceptibility, environmental triggers, and immune dysregulation. Heritability of HT is estimated at approximately 0.64, involving multiple HLA and non-HLA loci (5). Environmental factors such as iodine excess, selenium deficiency, interferon-α (IFN-α) exposure, and viral infection can disrupt thyroid immune tolerance against a genetically susceptible background. At the immune effector level, HT is characterized by Th1/Th17-biased immune responses, regulatory T cell (Treg) functional deficiency, and sustained production of autoantibodies (6). However, research has long centered on aberrant activation of the adaptive immune response, while the role of the thyroid follicular cell (hereafter thyrocyte) itself in this process has received comparatively less attention.

The endoplasmic reticulum (ER) is a central organelle in eukaryotic cells responsible for secretory protein folding, post-translational modification, and quality control (7). When the load of unfolded or misfolded proteins within the ER lumen exceeds its processing capacity, cells activate the unfolded protein response (UPR), which coordinates transcriptional reprogramming, translational attenuation, and ER-associated degradation (ERAD) through three signaling branches—PERK, IRE1α, and ATF6—to restore proteostasis (8). When adaptive UPR proves insufficient, sustained endoplasmic reticulum stress (ERS) shifts toward pro-apoptotic and pro-inflammatory programs, contributing to the pathogenesis of diverse diseases (9). In recent years, the regulatory roles of the UPR in immune cell differentiation, development, and effector function have attracted considerable attention, with its dysregulation implicated in autoimmune disorders, including rheumatoid arthritis, systemic lupus erythematosus, and inflammatory bowel disease (10). Thyrocytes possess a unique organ-specific vulnerability to ERS, since their continuous synthesis of structurally complex Tg keeps the ER near its folding limit even physiologically (11). Consistent with this, ERS markers are upregulated in AITD and HT patient tissue (12) and ERS pathways are activated in HT mouse models (13), suggesting that ERS may link environmental triggers, thyrocyte injury, and autoimmune activation.

Although the pathogenic roles of ERS in neurodegenerative diseases, metabolic disorders, and cancer have been systematically reviewed (14), its contribution to HT pathogenesis lacks comparable integration. Existing evidence is dispersed across three domains—thyroid cell biology, immune-cell UPR regulation, and thyroid autoimmunity—without a narrative that unifies target-organ injury and immune effector amplification within a coherent ERS framework. This review systematically organizes the above evidence, covering in sequence: the ER homeostatic foundation in thyroid physiology; triggers of ERS; multi-level ERS-mediated thyrocyte injury; UPR regulation of the HT immune microenvironment; and signaling intersections between ERS and other pathogenic mechanisms. For each component, evidence strength is stratified by proximity to HT/EAT pathology. Building on this synthesis, the Discussion section proposes an integrative, experimentally testable perspective and evaluates the translational prospects and current limitations of ERS-targeted therapy.

This review searched PubMed, Web of Science, and Scopus from database inception to April 2026 using search terms centered on endoplasmic reticulum stress, unfolded protein response, Hashimoto’s thyroiditis, autoimmune thyroiditis, thyrocytes, and immune cells, supplemented by manual citation tracing of key references. This is a narrative review; no formal risk-of-bias assessment or quantitative synthesis was performed. To enable readers to independently judge each piece of evidence’s proximity to HT pathology, the review classifies all mechanistic evidence by its source system rather than by evidence-based medicine study-design hierarchies: (i) direct evidence, defined as findings obtained from HT patient tissue or EAT/HT animal models; (ii) thyroid-cell evidence, defined as mechanistic findings derived from thyroid-derived cells or thyroid-specific models, typically under pharmacologically induced ERS; and (iii) extrapolated evidence, defined as mechanistic findings originating from non-thyroid experimental systems. Within each evidence category, the review further distinguishes causal findings—established through knockout, knockdown, pharmacological rescue, or temporal experiments—from correlative or descriptive findings. This classification reflects proximity to disease and causal strength and is not equivalent to a rating of study quality. Because direct HT/EAT evidence remains confined to a few unreplicated studies, the contribution of this review is to organize a scattered literature along the axes defined above and to recast the residual gaps as an explicit, testable research agenda.

## 2 Endoplasmic reticulum homeostasis in thyroid physiology

### 2.1 Thyroglobulin synthetic load and folding complexity

Thyrocytes depend critically on ER proteostasis because they continuously synthesize thyroglobulin (Tg), the predominant secretory protein and the precursor for hormone synthesis (15). Tg accounts for more than 75% of total thyroid protein, and over half of newly synthesized protein is Tg (16). Its folding is biochemically demanding: nascent Tg must undergo N-linked glycosylation, disulfide-bond formation, conformational maturation, and homodimerization, each requiring specific chaperones and oxidoreductases (17). Classic studies established that Tg folding proceeds through coordinated BiP, calnexin, and ERp57/PDI handoffs and through mixed-disulfide intermediates, and is acutely sensitive to ER luminal calcium (18–21). Because Tg is both highly abundant and structurally demanding, the thyrocyte ER operates near saturated folding load even under physiological conditions (22), and any perturbation exceeding this capacity may activate the UPR (23). This intrinsic vulnerability makes thyrocytes highly sensitive to factors that disturb ER proteostasis.

### 2.2 Continual dependence on ER proteostasis

A second layer of vulnerability comes from the continual demand for correctly matured Tg. Within the follicle, secreted Tg is iodinated at the apical membrane and re-internalized for lysosomal hormone release (24), and newly secreted Tg is preferentially used, so sustained hormone production depends on continual secretory-pathway supply rather than stored colloid alone (16). Hormone synthesis also requires TPO and DUOX-derived H_2_O_2_ at the apical membrane (25), while Tg maturation within the ER depends on disulfide-bond formation and oxidoreductase-assisted folding (17, 26); thyrocytes thus combine high-volume secretory output with strict redox dependence. Yu et al. validated this directly, showing in mouse thyroid organoids that excess iodide activates XBP1-mediated UPR and suppresses hormone-synthesis genes (27). Thyrocyte vulnerability therefore arises both from the difficulty of Tg folding and from a functional program requiring repeated supply of correctly matured Tg under tight redox control.

### 2.3 Functional consequences of ERS for thyrocytes

This vulnerability model has experimental support. In thyroid cell lines, pharmacological ERS inducers, such as tunicamycin and thapsigargin, activate all three UPR branches and elicit thyroid-specific functional impairments at sublethal intensities, indicating that functional suppression is not merely secondary to cell death (28–30). Under chronic ERS, thyrocytes can survive at the cost of differentiated function (31), and *in vivo*, ER quality-control defects in thyrocytes induce tissue injury and immune infiltration (32). Recent detection of upregulated ERS markers in AITD patient tissue and HT animal models (12, 13) connects this cell-biological vulnerability to autoimmune thyroid pathology; the molecular mechanisms of each injury mode are detailed in the following section.

Taken together, the physiological vulnerability of thyrocytes is supported primarily by thyroid-cell and thyroid-specific models, whereas its relevance to HT rests on correlative observations from HT/EAT tissues and animal models.

## 3 ERS in the pathogenesis of HT

### 3.1 Triggers of ERS in Hashimoto’s thyroiditis

Thyrocytes continually synthesize and fold large quantities of Tg under physiological conditions, with their ER persistently operating under high load. This intrinsic vulnerability renders thyrocytes highly sensitive to diverse environmental and endogenous stimuli. In the pathological context of HT, these triggers can disrupt proteostasis, activate the UPR, and contribute to cellular injury and autoimmunity. Established triggers include iodine excess, IFN-α, drugs such as amiodarone, oxidative stress, and epigenetic dysregulation.

#### 3.1.1 Iodine excess

Iodine intake shows a U-shaped relationship with thyroid autoimmune risk (33), and epidemiological data link iodine excess to rising autoantibody positivity and subclinical hypothyroidism, particularly in susceptible populations (34–36). Among the mechanisms, ERS may be a critical node. Excess iodide enhances Tg iodination, which alters Tg conformation, exposes cryptic epitopes, and may interfere with ER folding quality control and trigger the UPR (37). Excess iodide also drives DUOX-mediated H_2_O_2_ production beyond the needs of hormone synthesis, disrupting ER redox homeostasis (38). *In vitro*, abnormal iodine exposure activates all three UPR branches-PERK, IRE1α, and ATF6-in thyroid cells with upregulation of MCP-1 (39); in NOD.H2h4 mice, iodide supplementation accelerates spontaneous thyroiditis alongside impaired thyrocyte antioxidant capacity (40); and Zhou et al. confirmed upregulation of the ERS markers BiP, ATF6, p-IRE1α, and p-JNK in a Tg-plus-high-iodine HT mouse model (13). These effects act on an ER already operating near its folding limit, rendering the UPR readily activated.

#### 3.1.2 Interferon-α

During viral infection, IFN-α is a recognized environmental trigger of autoimmune thyroid disease, and up to 40% of patients on IFN-α therapy develop thyroid antibodies (41). In human thyroid cells, Faustino et al. showed that IFN-α raises Tg mRNA yet lowers Tg protein, and simultaneously activates all three UPR branches, as evidenced by coordinated elevation of BiP, XBP1s, ATF4, and CHOP expression. The Tg loss, however, proved autophagy/lysosomal rather than ERS-dependent: chemical chaperones reversed the UPR but did not restore Tg, whereas blocking lysosomal degradation did. In Graves’ disease and HT tissue, both UPR markers (BiP, CHOP, XBP1s) and autophagy markers (ATG5, LC3) were elevated relative to normal thyroid (42). The same study found that the ERS inducer thapsigargin upregulated IFN-α2 and MHC class I, pointing to a possible ERS-IFN-α vicious cycle. Acting on an ER already under high secretory load, IFN-α thus imposes a multi-pronged insult: it increases Tg transcription, accelerates Tg autophagic degradation, and activates the UPR.

#### 3.1.3 Pharmacological factor: amiodarone

Amiodarone, a commonly used antiarrhythmic agent, contains a high iodine content in its molecular structure and exhibits high accumulation and long half-life in thyroid tissue; thyroid dysfunction is its principal endocrine adverse effect (43). Lombardi et al. (2015) demonstrated in human thyroid cells that amiodarone directly induces ERS marker expression while downregulating Tg protein levels, and 4-PBA pretreatment completely abrogated these effects (44). Wen et al. further detected aberrant upregulation of XBP1s in HT patient tissue and demonstrated in thyrocytes that amiodarone aggravates ERS and apoptosis through a LINC00842/miR-214/FASL competing endogenous RNA regulatory axis; silencing XBP1s significantly suppressed amiodarone-induced apoptosis (45). This finding connects an ERS effector molecule to the Fas-mediated apoptotic mechanism characteristic of HT as a complete signaling pathway. The toxic effects of amiodarone can be understood as a multi-faceted assault on the intrinsic vulnerability of thyroid cells: on the one hand, its high iodine content may interfere with Tg folding; on the other, its direct activation of the UPR further undermines the cell’s proteostatic capacity, thereby driving apoptosis.

#### 3.1.4 Oxidative stress

The thyroid is one of the few organs where ROS are essential to normal function, so its follicular cells live in a persistently oxidative environment (46). ROS produced in excess of hormone-synthesis needs can propagate into the ER lumen and disrupt the redox balance required for disulfide-bond formation, directly triggering the UPR (47). DUOX-derived H_2_O_2_ supports TPO-catalyzed iodination (38) and is cleared largely by selenoprotein-dependent antioxidant defense (48); accordingly, selenium deficiency is associated with higher HT risk, with one cohort reporting a roughly threefold 6-year incidence in a low-selenium versus high-selenium region (49). Within the ER, disulfide-bond formation by ERO1α itself generates ROS (50), and superimposed exogenous oxidative stress disrupts luminal redox homeostasis and activates the UPR (51). The pro-apoptotic UPR effector CHOP (52) further upregulates ERO1α, establishing mutual amplification between oxidative stress and ERS (53). At the HT level, this coupling has only indirect support: Santos et al. linked SELENOS×NFE2L2 variants to HT risk and established a bidirectional Nrf2–SelS interaction in PCCL3 thyrocytes and mouse thyroid (54), but whether this antioxidant axis couples to the UPR in thyrocytes is untested.

#### 3.1.5 Epigenetic dysregulation

Recent research has revealed a critical role for epigenetic modifications in ERS regulation (55). Mo et al., using integrated single-cell, whole-transcriptomic, and metabolomic analyses, found increased ATF4-positive thyroid follicular epithelial cells with elevated ERS in AITD patient tissue (12). Mechanistically, the m6A reader hnRNPC modifies ATF4 mRNA, raising ATF4 expression at the transcriptional, processing, and translational levels. In thyroid cell lines, hnRNPC-driven ATF4 overexpression activated ERS signaling and induced apoptosis and necroptosis, a death program proposed to contribute to tissue destruction in HT, while inhibiting hnRNPC or ATF4 reduced cell death and delayed disease progression. This extends ERS regulation from protein-folding load into RNA epigenetics: even without overt folding abnormalities, m6A-driven ATF4 overexpression can activate pro-apoptotic ERS, and environmental factors acting through m6A may shift thyrocytes into an ERS state before classical protein-load increases.

Taken together, thyrocyte ERS in HT is unlikely to stem from a single factor. It more plausibly reflects the combined action of iodine disturbance, viral cytokine signaling, drug toxicity, redox imbalance, and epigenetic abnormality. These factors are themselves interconnected: iodine excess provokes oxidative stress and Tg conformational change; IFN-α and ERS/autophagy form a self-amplifying loop; oxidative stress and ERS amplify each other through the CHOP/ERO1α axis; and epigenetic change can activate the UPR independently of folding load. Each trigger acts on an ER already under high load, making thyrocytes prone to shift from adaptive UPR toward a pro-apoptotic, pro-immune ERS state that participates in the amplification and progression of autoimmune thyroid injury. The evidentiary basis is strongest for iodine excess and m6A dysregulation, both of which have direct HT/EAT support; IFN-α and amiodarone combine cell-line mechanistic dissection with patient-tissue marker validation; and the ERS link of oxidative stress rests mainly on extrapolation.

### 3.2 ERS-mediated thyrocyte injury

When intra- and extracellular triggers accumulate beyond the threshold of adaptive ER compensation, the UPR shifts from a protective to a destructive mode, inflicting multi-level injury upon thyrocytes. These injuries encompass apoptosis, defective Tg processing and secretion, suppression of thyroid-specific gene expression, and loss of epithelial phenotype. Given that thyrocytes critically depend on ER proteostasis to maintain their synthetic and secretory functions, ERS-induced injury may constitute the cytopathological basis for progressive loss of thyroid function. It should be noted that, with the exception of the single-cell data from Mo et al. derived from AITD patient tissue, current evidence is predominantly derived from *in vitro* cell models treated with pharmacological ERS inducers such as thapsigargin and tunicamycin, which may differ from the actual intensity, duration, and pathway activation patterns of ERS in HT *in vivo*, underscoring the need for validation in additional clinical-specimen studies.

#### 3.2.1 PERK-ATF4-CHOP axis-driven thyrocyte apoptosis

In general UPR biology, sustained ERS triggers apoptosis chiefly through the PERK-eIF2α-ATF4 axis: adaptive ATF4 targets support amino acid metabolism and redox balance (8, 56), but with persistent stress, ATF4 and CHOP drive a pro-apoptotic program in which CHOP suppresses BCL-2, induces BH3-only proteins, and promotes mitochondrial apoptosis, with parallel ERO1α/IP3R calcium and IRE1α-JNK routes (8, 53, 57). These cascades are established in non-thyroid cells, and their equivalence in HT thyrocytes remains to be validated. The HT-specific evidence is more limited but convergent. Mo et al. identified an ATF4-positive thyroid follicular epithelial subpopulation with apoptotic features in AITD tissue, and showed that hnRNPC- or ATF4-knockdown in thyroid cell lines reduced cell death (12). Zhou et al. detected elevated IRE1α and JNK phosphorylation in an HT mouse model, indicating that the IRE1α-JNK pathway is also activated *in vivo* (13).

#### 3.2.2 Thyroglobulin processing defects

Tg folding strictly depends on ER luminal chaperones and oxidoreductases, and any interruption causes Tg to be recognized as defective, retained, and degraded (17). Lombardi et al. found that amiodarone-induced ERS lowered Tg protein without changing Tg mRNA in human thyroid cells, and the proteasome inhibitor MG132 reversed this, confirming ERAD-mediated degradation as the principal mechanism (44). Beyond degradation, ERS also impairs Tg quality upstream: Wen et al. showed in FRTL-5 cells that ERS inhibits both N-linked glycosylation and phosphorylation of Tg (28), and defective glycosylation, critical for Tg solubility and quality-control recognition, leads to ER retention. Reduced mature Tg lowers the tyrosine residues available for iodination, which, if operative in HT *in vivo*, could contribute to reduced hormone synthetic capacity. All evidence here derives from cell lines under pharmacological ERS; no study has quantified Tg processing intermediates or ERAD activity in HT patient tissue.

#### 3.2.3 Broad suppression of thyroid-specific gene expression

ERS injury extends beyond Tg processing to broad transcriptional reprogramming. In tunicamycin-treated FRTL-5 cells, Wen et al. found reduced mRNA of NIS, TPO, and Tg, with the NIS decline impairing iodide uptake (28), and resveratrol reversed these effects, indicating a modulable causal link (29). The suppression centers on downregulation of the differentiation transcription factors TTF-1 (NKX2-1), FOXE1, and PAX-8. Ulianich et al. showed that as little as 30 minutes of thapsigargin or tunicamycin, without significant cell death, downregulated thyroid differentiation genes, indicating an active adaptation at sublethal ERS rather than a death consequence (30). Mechanistically, lowering transcription of high-load secretory genes reduces ER protein influx, an attempt to restore homeostasis that, under chronic ERS, persists at the cost of differentiation: Morishita et al. found that chronically misfolded-Tg cells remained viable but lost PAX8, FOXE1, and TPO (31). If sustained in the chronic inflammation of HT, this could progressively reduce hormone synthesis, consistent with HT-associated hypothyroidism, though no longitudinal study has confirmed the causal link. All evidence here is from pharmacologically induced cell lines, so the connection to hypothyroidism progression in HT remains a mechanistic inference.

#### 3.2.4 Dedifferentiation and mesenchymal phenotype switching

The thyroid follicle depends on a polarized epithelial monolayer for directional iodide transport and colloid storage (22). Ulianich et al. found that ERS, alongside suppressing differentiation genes, induced epithelial-mesenchymal transition features in PCCl3 cells: loss of E-cadherin (CDH1), cytoskeletal remodeling, and induction of vimentin and the EMT factor SNAI1 (30). In polarized FRT cells, the thyroid-specific cadherin CDH16, a PAX-8 target essential for apical-basal polarity and lumen formation, was downregulated (58). Linking these to *in vivo* pathology, Zhang et al. observed follicular collapse and loss of epithelial integrity in a non-autoimmune mutant-Tg ERS mouse model, with damaged regions becoming sites of bone marrow-derived macrophage infiltration (32); ERS-driven dedifferentiation and architectural disruption may thus be a candidate mechanism linking thyrocyte injury to immune recruitment. EMT may also promote the interstitial fibrosis seen in late HT, though this is not directly validated.

Current evidence directly supports ERS activation in HT thyrocytes at the cellular level (Mo et al., n = 2), with additional corroboration in thyroid tissue (Zhou et al.). However, the four injury modes derive almost entirely from cell-line or general UPR biology; the extent to which each is ERS-driven in HT *in vivo* awaits direct validation.

#### 3.2.5 Signal transmission from thyrocyte injury to the immune system

For the above injury modes to play an actual role in HT pathogenesis, they must be recognizable by the immune system, potentially through the following routes. One pathway may involve the release of damage-associated molecular patterns (DAMPs). In AITD patients, Peng et al. detected significantly elevated serum HMGB1 and HSP60 levels relative to healthy controls, both positively correlated with TPOAb and TgAb titers; in the same study, patient PBMCs showed upregulated TLR2, TLR3, and TLR9 expression with enhanced downstream MyD88 signaling (59). Thyrocytes themselves express multiple TLRs and can recognize endogenous DAMPs to participate in initiating innate immune responses (60). However, these DAMPs are not specific products of ERS. HMGB1 and HSPs can be released by diverse forms of cell death—apoptosis, necrosis, pyroptosis, and others—while HT thyrocytes simultaneously face direct immune-cell killing and multiple death programs. The critical gap in this pathway is that DAMPs detected in HT lesional tissue cannot currently be attributed to ERS.

Another pathway involves altered autoantigen immunogenicity, which can occur through two mechanistically independent routes. The first is iodination-dependent modification. Iodination can expose normally cryptic epitopes on the Tg molecule, with iodinated Tg eliciting stronger T cell proliferative responses than non-iodinated Tg (37). This mechanism has been discussed in the context of the iodine-excess hypothesis of HT and is characterized by modification driven by iodine itself, independent of ERS. The second route is ERS-dependent defective post-translational modification. ERS can inhibit N-linked glycosylation and phosphorylation of Tg (28), resulting in folding and maturation abnormalities. In other autoimmune diseases, aberrant post-translational modifications generating neo-autoepitopes and driving autoimmune responses are well established: in rheumatoid arthritis, modifications such as citrullination generate neo-epitopes on self-proteins, inducing anti-modified protein antibodies (AMPAs) that serve both as diagnostic markers and central pathogenic mediators (61); in IgA nephropathy, aberrant glycosylation (galactose deficiency) of the IgA1 hinge-region O-glycans exposes neo-epitopes and induces specific autoantibody production, driving nephritogenic immune-complex deposition (62). Two boundaries must be explicitly recognized: whether ERS-induced Tg glycosylation/phosphorylation defects similarly expose neo-epitopes has not been directly validated in either HT or any other thyroid model; and unlike iodination, the source of modification in this mechanism is ERS rather than iodine, making these two epitope-generating pathways independent and potentially additive.

### 3.3 UPR regulation of the Hashimoto’s thyroiditis immune microenvironment

The role of ERS in HT pathogenesis is not confined to target-organ injury. Increasing evidence indicates that the three UPR signaling branches play critical regulatory roles in the differentiation, function, and survival of both adaptive and innate immune cells. The immunopathological hallmarks of HT include Th1/Th17-dominant polarization, Treg functional deficiency, sustained autoantibody secretion by plasma cells, and pro-inflammatory activation of macrophages. This section systematically reviews the regulatory mechanisms of individual UPR branches on these immune cell subsets and, on this basis, explores the positive-feedback relationship between ERS-mediated HT target-cell injury and immune effector amplification. It must be explicitly noted that, with the exception of the EAT model study by Ding et al., the vast majority of immune-cell UPR regulatory evidence cited in this section derives from non-HT experimental systems; its precise role in HT immunopathology awaits validation.

#### 3.3.1 UPR regulation of T cell subset differentiation

HT is characterized by Th1- and Th17-biased immune responses (63), and several UPR components regulate these subsets in non-HT systems. The PERK-eIF2α-ATF4 axis supports Th1 differentiation: in ATF4 conditional-knockout mice, ATF4-deficient CD4^+^ T cells show impaired IFN-γ production under Th1 polarization yet enhanced Th17 responses in EAE, indicating divergent control of the two subsets (64). The IRE1α-XBP1s axis conversely promotes Th17 differentiation under cellular stress, and its inhibition attenuates Th17 expansion and EAE severity (65). Treg functional deficiency is an important component of HT pathogenesis (66). Treg-specific Hrd1 deletion elevates IRE1α activity and suppresses FoxP3, causing Treg dysfunction only under inflammatory stimulation, with no spontaneous thyroiditis in knockout mice (67); this inflammation-dependence is relevant to HT, where sustained TNF-α and IFN-γ may constitute the conditions that destabilize FoxP3. In rheumatoid arthritis and SLE, destabilized FoxP3^+^ Tregs can transdifferentiate into IL-17-producing exFoxp3 Th17 cells (68, 69); whether this Treg-Th17 plasticity operates in HT is a logical but untested hypothesis.

#### 3.3.2 Plasma cell differentiation and sustained autoantibody production

The IRE1α-XBP1s axis is a key pathway governing plasma cell differentiation and immunoglobulin secretion (70). During terminal B cell-to-plasma cell differentiation, the transcriptional repressor Blimp-1 induces XBP1 expression, and XBP1s promotes the transcription of secretory-pathway genes, expanding ER area and increasing ribosome numbers (71). A notable feature of the plasma cell UPR is its asymmetry: the IRE1α-XBP1s pathway is selectively activated, while PERK signaling is actively suppressed (72). B cell-specific XBP1-knockout mice exhibit markedly reduced IgM secretion without affecting B cell survival or proliferation (73). In HT tissue, two correlative signals are consistent with this axis. Avramidou et al. found interstitial plasma cells with markedly dilated rough ER on TEM (74), and Mo et al. reported enrichment of ER protein-processing pathways across B-plasma cell subsets by scRNA-seq (12). Neither, however, distinguishes pathological ERS from the high secretory load normal to professional antibody-secreting cells, and neither resolves specific UPR branches, provides functional validation, or links the signal to sustained autoantibody production. HT plasma cell ERS therefore rests on transcriptomic and morphological correlation alone; its pathological nature and functional consequences await confirmation.

#### 3.3.3 Macrophage polarization and pro-inflammatory activation

Macrophages represent the immune cell type with the highest level of evidence within the HT/EAT system in this section, possessing both correlative evidence from HT tissue and pharmacological functional support from EAT models. Ding et al. observed a significantly increased proportion of M1 macrophages in HT patient thyroid tissue, accompanied by STING pathway activation and elevated oxidative stress; immunofluorescence co-localization further revealed that upregulation of the ERS marker IRE1α was found not only in thyrocytes but also localized to M1-type macrophages, with no similar changes observed in M2 macrophages (75). In EAT rats, mesenchymal stem cell treatment inhibited STING, lowered thyroid ERS and NLRP3, and reduced IRE1α and M1 polarization, while the STING agonist DMXAA reversed these effects. IRE1α was only detected, not manipulated, so its causal necessity for M1 polarization remains unestablished. Supporting mechanism comes from non-HT macrophage biology: TLR2/TLR4 activate IRE1α-XBP1s, which drives sustained IL-6 and TNF transcription (76), and ERS-activated IRE1α can additionally engage NF-κB to promote TNF-α, IL-1β, and IL-6, further reinforcing the macrophage pro-inflammatory phenotype (77). In HT, DAMPs from tissue injury could in principle sustain this program, and the resulting cytokines could re-induce thyrocyte ERS, but such transcellular propagation has no direct validation.

#### 3.3.4 Chemotactic recruitment of immune cells to the thyroid

The immune-cell effects above depend on recruitment to the thyroid, for which direct HT evidence exists. Under IFN-γ and TNF-α, thyrocytes secrete CXC chemokines; CXCL10 recruits Th1 cells via CXCR3, and these cells release further IFN-γ and TNF-α, forming a self-amplifying recruitment loop (78, 79). CXCL9 and CXCL10 are elevated in aggressive autoimmune thyroiditis (80), and stromal ACKR1^+^ endothelial and CCL21^+^ fibroblast subsets help build a lymphocyte-permissive niche (6). Whether ERS drives this loop is untested: UPR regulation of chemokines in non-thyroid models is context-dependent and even bidirectional (81), and no study has examined ERS-driven chemokine secretion by thyrocytes in HT or EAT. This step remains a candidate awaiting direct testing.

#### 3.3.5 Potential ERS regulation of dendritic cell antigen presentation

Dendritic cells present autoantigen and activate T cells in HT (82), but UPR evidence for DC function is confined to non-HT systems. DC-specific XBP1 deletion impairs CD8α^+^ cDC cross-presentation (83), and in the tumor microenvironment, XBP1 activation driven by lipid peroxidation products abolishes DC antigen-presenting function, which XBP1 deletion restores (84). Because the HT thyroid microenvironment likewise carries oxidative stress and lipid peroxidation, the activation state of the IRE1α-XBP1 axis in infiltrating DCs is a clear entry point for testing this candidate mechanism.

**Figure 1:**
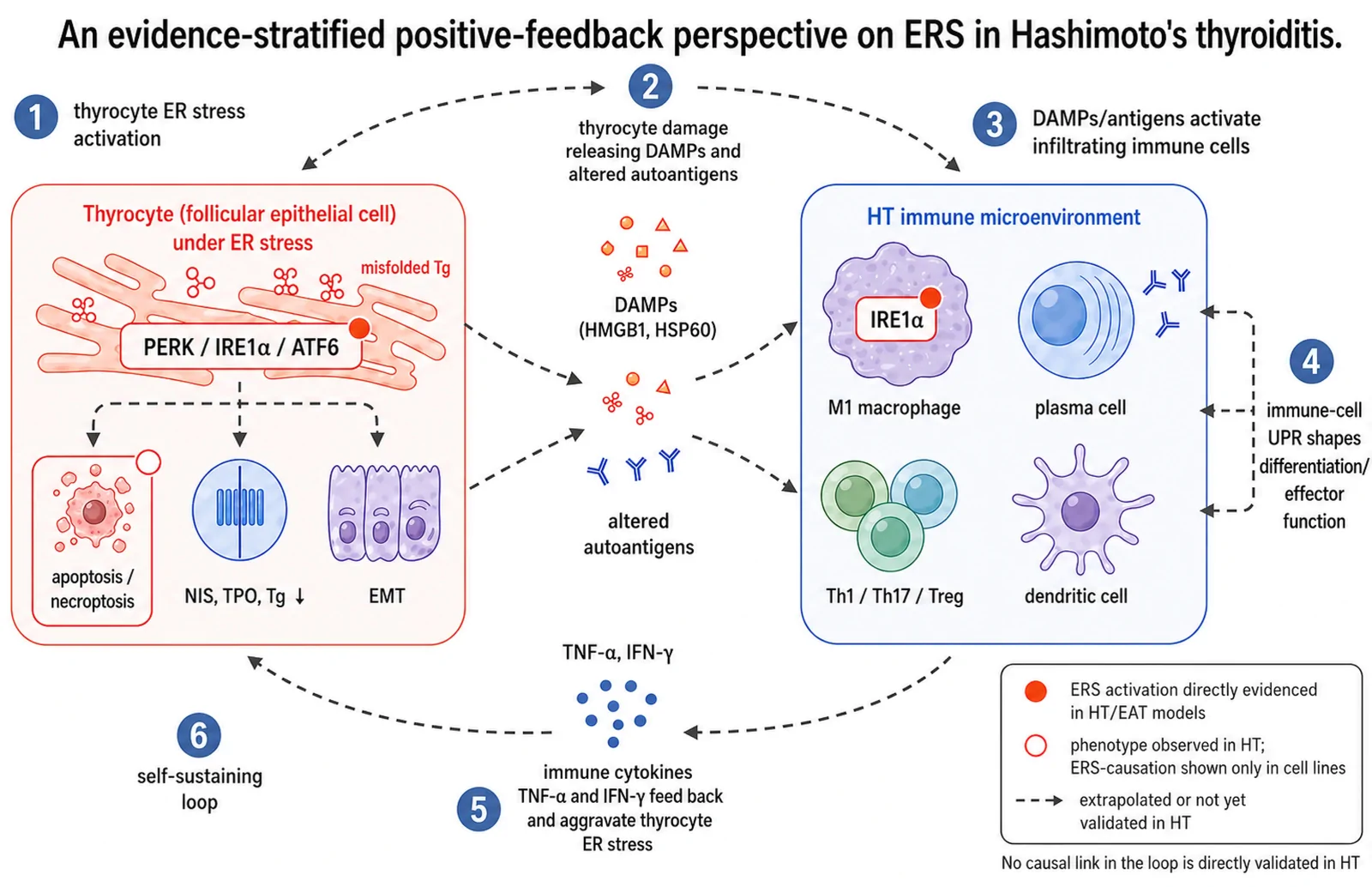
A hypothesized self-sustaining loop (Steps 1–6) linking thyrocyte ERS to immune effector amplification, presented as a framework for organizing evidence and exposing gaps rather than as an established pathway. Node markers encode HT-specific evidence at two levels. A filled dot marks the two nodes with direct evidence of ERS activation in HT or HT/EAT models: thyrocyte ER-stress activation (Step 1; PERK/IRE1α/ATF6; refs 12, 13) and the macrophage IRE1α component of Step 4 (ref 75). An open dot marks thyrocyte apoptosis/necroptosis (PDCD5, p-MLKL; ref 12), a death phenotype directly observed in HT tissue but whose causal dependence on ERS has been shown only in thyroid cell lines. Unmarked nodes (NIS/TPO/Tg suppression, EMT, plasma cell, T cell, dendritic cell) have no direct HT evidence of ERS involvement. All arrows are dashed, denoting relationships extrapolated from non-thyroid systems or not yet directly validated in HT; accordingly, no causal link in the loop has been directly validated in HT. EAT, experimental autoimmune thyroiditis; EMT, epithelial–mesenchymal transition; DAMPs, damage-associated molecular patterns; UPR, unfolded protein response.

Across immune-cell compartments, the evidence is strongest for macrophage UPR activation in HT/EAT, with IRE1α localized to M1 macrophages in HT tissue, though its causal necessity remains untested. Among the remaining subsets, only the plasma cell lineage adds HT scRNA-seq correlation, while T cell and dendritic cell evidence is entirely non-HT. Whether thyrocyte ERS injury and these immune mechanisms form a self-sustaining positive-feedback loop is assessed in the Discussion (**Figure 1**).

## 4 ERS and downstream effectors: potential crossroads

The thyrocyte injury mediated by ERS, as described above, does not represent an isolated endpoint. Four downstream pathological processes—mitochondrial dysfunction, autophagy dysregulation, pyroptosis, and ferroptosis—have each been independently observed in HT thyroid tissue or EAT models, and the molecular pathways through which ERS drives these four processes have been established in non-thyroid systems. However, whether ERS actually drives these downstream processes in HT lacks direct validation in every case. This section sequentially reviews the current state of evidence and gaps for each cross-mechanism.

### 4.1 Mitochondrial dysfunction

Avramidou et al., in TEM studies of HT patient thyroid specimens, demonstrated widespread mitochondrial swelling with poorly defined cristae in thyrocytes, coexisting with dilated ER cisternae (74); earlier ultrastructural pathology studies similarly documented concurrent ER and mitochondrial damage (85). Esfahanian et al. detected significantly increased mitochondrial DNA copy number and elevated levels of the oxidative damage marker 8-OHdG in the peripheral blood of HT patients, suggesting that mitochondrial dysfunction is not confined to the thyroid gland locally (86). In general UPR biology, the ER and mitochondria form physical contacts through mitochondria-associated ER membranes (MAMs), where PERK and IRE1α mediate inter-organellar Ca²^+^ and redox signal transmission, and MAMs can also serve as assembly platforms for the NLRP3 inflammasome (87–91). The morphological and peripheral blood molecular marker changes documented in HT are directionally consistent with the expected phenotype of MAM dysfunction, but no study has examined MAMs-associated protein expression changes in HT thyroid tissue, and the causal relationship between mitochondrial damage and ERS in HT has not been established.

### 4.2 Autophagy dysregulation

Zheng et al., using immunohistochemistry on HT patient thyroid tissue, detected significantly reduced LC3B protein expression in thyrocytes relative to healthy controls (92), whereas Faustino et al. detected elevated mRNA levels of the autophagy markers ATG5 and LC3 in AITD patient tissue (42). The apparent contradiction between decreased protein and increased mRNA may reflect impaired autophagic flux rather than simple activation or inhibition—that is, upregulation of autophagosome formation signals coupled with blockade of the lysosomal degradation step. In general UPR biology, all three UPR branches can independently initiate autophagy (93); in thyroid-derived cells, Zhang et al., using thyrocyte-specific gene-knockout models, demonstrated that ERAD—rather than autophagy—is the principal protective mechanism for ER proteostasis (32). Tseng et al. found that T3 can enhance lysosomal activity through LAMP2 upregulation to resist ERS-induced cell death (94), which carries particular significance for HT: impaired T3 synthesis in HT thyrocytes may lead to decreased LAMP2 expression and weakened lysosomal function, with impaired autophagic clearance further exacerbating ERS. However, the causal direction and temporal sequence between ERS and autophagy dysregulation have not been resolved in the HT/EAT system.

### 4.3 Pyroptosis

NLRP3-mediated pyroptosis has been amply confirmed in HT thyroid tissue. Guo et al. detected upregulation of four inflammasomes, including NLRP3 in HT patient tissue, with NLRP1 and ASC positively correlated with serum autoantibody titers (60); Zhao et al. demonstrated that USP1 enhances pyroptosis by deubiquitinating and stabilizing NLRP3/ASC/caspase-1, and the USP1 inhibitor ML323 ameliorated pathology in an HT mouse model (95). In general UPR biology, ERS can activate the NLRP3 inflammasome through two pathways: the IRE1α-TXNIP axis and the PERK-NF-κB axis (96). However, ERS-mediated NLRP3 activation through these two pathways has not been directly validated in HT thyroid tissue.

### 4.4 Ferroptosis

Sacristán-Gómez et al. detected decreased GPX4 and elevated lipid peroxidation marker 4-HNE in HT patient thyroid tissue (97); Mou et al. demonstrated in EAT rats that ursolic acid ameliorated pathology and restored Th17/Treg balance by upregulating GPX4 and SLC7A11 (98). In general UPR biology, the PERK-ATF4-CHAC1 pathway can trigger ferroptosis by upregulating CHAC1 to catalyze GSH degradation and deplete GPX4 substrate (99, 100). However, direct evidence of PERK-ATF4-CHAC1 pathway activation in HT thyroid tissue remains absent.

For these downstream phenotypes, direct HT/EAT observations establish their occurrence, whereas the proposed causal linkage to ERS remains unvalidated in HT.

## 5 Therapeutic prospects and their limitations

All current ERS-targeted interventions for HT remain at the preclinical stage or are based on indirect mechanistic associations; no strategy has yet been validated as an ERS-stratified HT therapy. The existing evidence and its limitations are critically reviewed below.

### 5.1 Direct ERS suppression by chemical chaperones

4-PBA binds to the hydrophobic regions of unfolded proteins, assisting their correct folding and reducing the burden of unfolded protein within the ER lumen, thereby suppressing excessive UPR activation (101). Zhou et al. found elevated BiP, ATF6, p-IRE1α, and p-JNK in both the thyroid and adipose tissue of euthyroid HT mice; 4-PBA suppressed adipose p-IRE1α and p-JNK and lowered serum TNF-α and IL-6, though its effect on thyroid ERS markers was not directly tested (13). This study provided preliminary demonstration of the potential of 4-PBA to alleviate ERS in the HT model. At the *in vitro* level, Chen et al. demonstrated that in Nthy-ori 3–1 thyrocytes treated with abnormal iodine concentrations, PERK, IRE1α, and ATF6 expression was upregulated, and 4-PBA intervention reversed this ERS activation and suppressed downstream P38/MAPK pathway-mediated MCP-1 expression (39).

However, 4-PBA as a preclinical ERS modulator faces barriers to clinical application in HT. 4-PBA is currently approved only for urea cycle disorders, and its evidence in the HT field is confined to the aforementioned animal models and *in vitro* experiments, with no human trial data. As a non-specific chemical chaperone, 4-PBA’s protein binding lacks target selectivity, and chronic systemic administration may interfere with normal protein-folding quality control and trigger off-target effects. Notably, 4-PBA also acts through the ERS pathway in HT-relevant extrathyroidal settings, reversing TSH-induced insulin resistance and adipose M1 polarization in a subclinical hypothyroidism model (102, 103) and correcting the DIO2 Thr92Ala brain phenotype by restoring D2 folding (104); its clinical advancement still depends on thyroid-targeted delivery and HT-specific safety evaluation. The endogenous bile-acid chaperone TUDCA suppresses ERS in metabolic and neurodegenerative models (105, 106) but has scarcely been tested in HT or thyrocyte ERS.

### 5.2 Selenium supplementation: indirect modulation of ER proteostasis

The thyroid has among the highest selenium content per tissue mass, and several of the ER-resident selenoproteins, including SELENOS and SELENOK, act within the ERAD complex to clear misfolded proteins (107, 108), giving selenium a plausible link to ER proteostasis. Clinical evidence is comparatively developed: a meta-analysis of 35 RCTs found that selenium supplementation lowered TSH, TPOAb, and malondialdehyde in HT patients not on levothyroxine, without excess adverse events (109). From an ERS-translation standpoint, however, three problems remain. First, durability and clinical endpoints are uncertain: the effect was lost by 12 months in one systematic review (110), a large 2024 RCT found no benefit for thyroid-specific quality of life (111), and data on hypothyroidism progression or ultrasound morphology are absent. Second, the safety window is narrow, since selenium follows a U-shaped dose-response and excess raises type 2 diabetes risk (112). Third, the ERS pathway itself is unvalidated in HT tissue: no study has examined the correlation between selenoprotein expression and ERS markers in HT thyroid, leaving the mechanistic rationale unconfirmed.

### 5.3 Emerging molecular targets and cell therapy

Specific nodes in the ERS pathway also offer precise targets. From the m6A-ATF4 mechanism described above, hnRNPC and ATF4 emerge as candidate targets, with knockdown of either reducing thyroid follicular epithelial cell death in AITD models (12); translation faces tissue-specific delivery of RNA-modification regulators, off-target silencing, and compensatory remodeling of the m6A network. Mesenchymal stem cells offer a cell-therapy route: in the EAT model, they lowered thyroid ERS and shifted macrophages away from the M1 phenotype (75), and although MSCs are already in clinical trials for other autoimmune diseases (113), HT evidence rests on a single animal study.

Among candidate interventions, 4-PBA has the strongest preclinical support. It suppresses ERS in abnormal-iodine thyroid-cell models. In HT mice, it reduced adipose ERS and alleviated systemic inflammation, but thyroid ERS was not assessed. Selenium has clinical support but lacks mechanistic validation, whereas hnRNPC/ATF4 and MSC approaches remain at the preclinical proof-of-concept stage. The shared bottleneck is the absence of HT-specific biomarkers to select patients likely to benefit from ERS intervention, and of non-invasive tools to monitor local thyroid ERS. Accordingly, ERS-targeted interventions should be regarded as investigational and are not ready for routine clinical practice in HT.

## 6 Discussion

Sections 2–5 above have respectively examined the current evidence for ERS in HT pathogenesis from five dimensions: the ER homeostatic foundation of thyrocytes, ERS triggers and their injurious effects, UPR regulation of the immune microenvironment, potential intersections with downstream effector mechanisms, and prospects for therapeutic translation. This section integrates these dispersed lines of evidence to answer four questions: Which mechanistic steps have been directly established in HT/EAT systems? Which remain merely reasonable extrapolations? Where does ERS sit within the causal chain of HT? And if these steps are integrated into a ‘thyrocyte injury–immune effector amplification’ positive-feedback perspective, what are the evidence gaps for each step, and how should they be experimentally tested?

### 6.1 Evidence directly established

**Table 1** lists, for each mechanistic step, the representative studies, experimental system source, principal findings, and HT-specific unresolved questions. Of the six steps of the positive-feedback perspective, only two have direct support from HT/EAT systems. For thyrocyte ERS activation (Step 1), Mo et al. localized the ERS signal to an ATF4-positive follicular epithelial subpopulation in HT patient tissue, with causal validation of hnRNPC/ATF4 knockdown done in thyroid cell models (12); Zhou et al. gave independent *in vivo* corroboration, though the marker changes were measured at the whole-thyroid level and could not be localized to thyrocytes versus infiltrating immune cells (13). For macrophage UPR activation (Step 4), Ding et al. localized IRE1α to M1 macrophages in HT tissue, and in EAT, the IRE1α signal and M1 polarization fell together under MSC treatment and rose with DMXAA; IRE1α was only detected, not manipulated, so its causal necessity is unestablished (75). At the trigger and therapeutic levels, iodine excess, m6A abnormality, and 4-PBA have direct HT/EAT evidence, while IFN-α and amiodarone combine cell-line dissection with patient-tissue markers (13, 42, 44, 45). All remaining steps lack direct HT/EAT evidence (**Table 1**); apoptosis/necroptosis, although directly observed in HT tissue (12), is attributed to ERS only on cell-line evidence.

**Table 1:** ERS-related mechanistic evidence in Hashimoto’s thyroiditis (HT).

Mechanism	Study	System & evidence tier		Evidence type	Key findings	Unresolved in HT
Thyrocyte ERS activation and triggers (Section 3.1)						
Iodine excess activates ERS in thyrocytes	Zhou et al., 2025 (13); Chen et al., 2019 (39)		Nthy-ori 3–1 cells [thyroid-cell]; HT mouse, Tg + high-iodine [HT/EAT]	Causal (cell line); Correlative (HT model)	PERK/IRE1α/ATF6 up *in vitro*, reversed by 4-PBA and SB203580 (causal); BiP/ATF6/p-IRE1α/p-JNK up in HT-mouse thyroid, 4-PBA reversal demonstrated in adipose, not thyroid	No human-tissue dose-response; single animal study, *in-vivo* ERS markers measured at whole-tissue level (Western blot), not localized to thyrocytes
IFN-α triggers ERS and Tg degradation	Faustino et al., 2018 (42)		Human thyroid cells *in vitro* [thyroid-cell]; HT/GD patient tissue [HT/EAT]	Causal (cell line); Correlative (patient tissue)	BiP, XBP1s, ATF4, CHOP up; Tg degraded via autophagy/lysosome; patient tissue (mixed HT/GD) showed elevated UPR and autophagy markers	Not tested in EAT animal model *in vivo*
Amiodarone induces ERS-dependent apoptosis	Lombardi et al., 2015 (44); Wen et al., 2022 (45)		Human thyroid cells [thyroid-cell]; HT patient tissue [HT/EAT]	Causal (cell line); Correlative (patient tissue)	ERS markers induced; Tg reduced via ERAD; 4-PBA blocked ERS; XBP1s silencing reduced apoptosis (LINC00842/miR-214/FASL); XBP1s elevated in HT tissue	No EAT/HT animal-model validation
Oxidative stress (NRF2–SELENOS antioxidant axis)	Santos et al., 2019 (54)		HT patient cohort + tissue, genetic association + IHC [HT/EAT]; PCCL3 cells, mouse thyroid [thyroid-cell]	Correlative (HT genetic association + IHC); Causal (Nrf2–SelS, PCCL3/mouse)	SELENOS × NFE2L2 variants modify HT risk (n=997); bidirectional Nrf2–SelS interaction shown in PCCL3/mouse thyroid; SelS reduced in HT follicular epithelium (IHC)	SelS/NRF2 antioxidant axis not linked to UPR activation in any thyroid system
m6A modification drives ATF4-mediated ERS	Mo et al., 2023 (12)		AITD patient tissue (2 HT, 2 GD, 1 control; scRNA-seq, multi-omics) [HT/EAT]; thyroid cell lines, knockdown [thyroid-cell]	Correlative (patient tissue); Causal (cell line)	ATF4^+^ thyrocytes increased in AITD tissue; hnRNPC enhanced ATF4 via m6A; hnRNPC/ATF4 knockdown reduced cell death	Single study (n=2 HT); replication in independent HT cohort needed
ERS-mediated thyrocyte damage (Section 3.2)						
PERK-ATF4-CHOP apoptosis	Mo et al., 2023 (12); Zhou et al., 2025 (13); Han et al., 2013 (53)		AITD patient tissue (2 HT, 2 GD, 1 control), HT mouse [HT/EAT]; non-thyroid cells, general UPR biology [non-thyroid]	Correlative (HT tissue); Causal (cell-line knockdown); Causal (non-thyroid)	ATF4^+^ thyrocytes elevated in AITD tissue; apoptosis/necroptosis markers elevated in HT thyrocytes; ERS→cell death causal via hnRNPC/ATF4 knockdown (cell line); IRE1α–JNK activated in HT mouse; CHOP cascade from general UPR biology	CHOP-driven mitochondrial apoptosis not validated in HT thyrocytes
Tg processing failure and ERAD degradation	Wen et al., 2017 (28); Lombardi et al., 2015 (44)		Human thyroid + FRTL-5 cells, pharmacological ERS [thyroid-cell]	Causal (cell line)	Tg protein decreased without mRNA change; MG132 confirmed ERAD; ERS inhibited Tg glycosylation and phosphorylation	No Tg processing-intermediate or ERAD-activity data from HT tissue
Thyroid gene suppression (NIS, TPO, Tg, PAX8, TTF-1)	Wen et al., 2017 (28); Wen et al., 2021 (29);Ulianich et al., 2020 (30); Morishita et al., 2020 (31)		FRTL-5, PCCl3 cells, acute + chronic ERS [thyroid-cell]	Causal (cell line)	Sublethal ERS suppressed differentiation genes and transcription factors; chronic ERS preserved viability at the cost of differentiation; resveratrol partially rescued	No longitudinal link to hypothyroidism progression in HT
EMT and dedifferentiation	Ulianich et al., 2020 (30); Zhang et al., 2023 (32)		PCCl3/FRT cells, thyrocyte-specific multi-defect mouse (*in vivo*, non-autoimmune) [thyroid-cell]	Causal (cell line); Causal (non-autoimmune mouse)	E-cadherin loss, vimentin/SNAI1 up, CDH16 down; combined ER-proteostasis defects caused follicular collapse with macrophage infiltration	EMT markers not examined in HT patient thyroid tissue
UPR in immune cells of the HT microenvironment (Section 3.3)						
ATF4 in Th1 and XBP1 in Th17 differentiation	Yang et al., 2018 (64); Brucklacher-Waldert et al., 2017 (65)		ATF4 conditional-KO mice, XBP1 inhibition, EAE model [non-thyroid]	Causal (non-thyroid autoimmune model)	ATF4-null CD4^+^ T cells: impaired Th1, enhanced Th17; XBP1 inhibition reduced Th17 expansion and EAE severity	No UPR data from HT thyroid-infiltrating T cells
Hrd1-IRE1α axis in Treg stability	Xu et al., 2019 (67)		Treg-specific Hrd1 conditional-KO mice [non-thyroid]	Causal (non-thyroid model)	Hrd1 loss → IRE1α accumulation, p38-mediated FoxP3 suppression under inflammation; IRE1α-kinase inhibitor rescued Tregs; no spontaneous thyroiditis in KO	UPR status of HT-infiltrating Tregs unknown
Treg–Th17 plasticity (FoxP3 instability)	Komatsu et al., 2014 (68); Jung et al., 2017 (69)		Autoimmune-arthritis mouse, human RA/SLE studies [non-thyroid]	Causal (non-thyroid model); Correlative (human autoimmune)	FoxP3-unstable Tregs convert to IL-17-producing exFoxp3 Th17 cells and contribute to pathology	ERS link to Treg-Th17 conversion not established in any system; unstudied in HT
IRE1α-XBP1s in plasma-cell function	Mo et al., 2023 (12); Nutt et al., 2015 (71); Todd et al., 2009 (73); Avramidou et al., 2023 (74)		B cell-specific XBP1-KO mice [non-thyroid]; HT thyroid tissue, TEM + scRNA-seq, n=2 HT [HT/EAT]	Causal (non-thyroid model); Descriptive (HT ultrastructure); Correlative (HT scRNA-seq, pathway-level)	XBP1s drove secretory-apparatus expansion; XBP1-KO reduced IgM secretion; HT plasma cells showed expanded rough ER (morphology, not functional UPR); HT plasma-cell subsets enriched for “protein processing in ER” pathway (scRNA-seq; not IRE1α-XBP1s-resolved)	No functional UPR data from HT thyroid-infiltrating plasma cells; transcriptomic enrichment is pathway-level only — cannot separate physiological secretory load from pathological ERS, nor resolve IRE1α-XBP1s
Chemotactic recruitment of immune cells to the thyroid	Rotondi et al., 2013 (78); Antonelli et al., 2014 (79); Antonelli et al., 2011 (80); Junjappa et al., 2018 (81)		Human thyrocytes, IFN-γ/TNF-α + AITD patient sera/tissue [HT/EAT]; non-thyroid autoimmune models, ERS–chemokine link [non-thyroid]	Causal (thyrocyte chemokine induction); Correlative (AITD patients); Causal (non-thyroid; bidirectional)	IFN-γ/TNF-α induce thyrocyte CXCL10 → CXCR3-mediated Th1 recruitment forming a self-amplifying loop; CXCL9/CXCL10 elevated in aggressive AITD; IRE1α-XBP1s regulates chemokines in non-thyroid models with direction-dependent effects	Whether ERS/UPR drives thyrocyte chemokine secretion not tested in HT or EAT
IRE1α in M1 macrophage polarization	Ding et al., 2024 (75)		HT patient tissue, EAT rat model, MSC therapy [HT/EAT]	Correlative (HT tissue, M1 co-localization); Correlative (EAT; IRE1α tracked MSC/STING manipulation, not itself perturbed)	HT thyroid: IRE1α/UPR markers co-localized to M1 (not M2) macrophages, with STING activation and elevated ER/oxidative stress; EAT: MSC lowered M1 IRE1α and M1 polarization, reversed by STING agonist DMXAA	causal role in M1 polarization untested; single study, no independent replication
XBP1 in dendritic-cell cross-presentation	Osorio et al., 2014 (83); Cubillos-Ruiz et al., 2015 (84)		DC-specific XBP1 conditional-KO mice, tumor microenvironment [non-thyroid]	Causal (non-thyroid model)	XBP1 loss impaired CD8α^+^ DC cross-presentation via tapasin mRNA decay; lipid-peroxidation-driven XBP1 disrupted DC antigen presentation in tumors	No data on UPR pathway status in HT-infiltrating DCs
Mitochondrial dysfunction (Section 4.1)						
Mitochondrial dysfunction in HT	Avramidou et al., 2023 (74); Esfahanian et al., 2021 (86)		HT patient thyroid tissue, TEM + peripheral blood, qPCR [HT/EAT]	Descriptive (HT tissue, TEM); Correlative (HT blood, qPCR)	TEM: mitochondrial swelling and cristae disorganization with ER-pool expansion in HT thyrocytes; blood: increased mtDNA copy number, elevated 8-OHdG	No MAMs-protein data; ERS–mitochondria causality untested in HT
Autophagy dysregulation (Section 4.2)						
Autophagy dysregulation in HT thyrocytes	Faustino et al., 2018 (42); Zheng et al., 2018 (92)		HT patient tissue, IHC + HT/GD tissue, mRNA [HT/EAT]; Nthy-ori 3–1 cells [thyroid-cell]	Correlative (patient tissue); Causal (cell line)	LC3B protein decreased in HT thyrocytes (IHC); IL-23 inhibited LC3B-II via AKT/mTOR/NF-κB (rescued by rapamycin/IKK-16/anti-IL-23); ATG5/LC3 mRNA elevated in AITD tissue, suggesting flux impairment	ERS–autophagy direction untested in HT/EAT
ERAD vs. autophagy in thyroid ER proteostasis	Zhang et al., 2023 (32); Tseng et al., 2023 (94)		Thyrocyte-specific Hrd1-KO + Atg7-KO mice [thyroid-cell]; rat liver, GC cells [non-thyroid]	Causal (thyroid-specific non-autoimmune model); Causal (non-thyroid)	Hrd1-KO with misfolded Tg caused follicular death and macrophage infiltration; Atg7-KO did not (ERAD dominant); T3 upregulated LAMP2, protecting against ERS death	Not HT/EAT models; T3–LAMP2 untested in HT hypothyroidism
Pyroptosis (Section 4.3)						
ERS-driven NLRP3 pyroptosis	Guo et al., 2018 (60); Zhao et al., 2024 (95); Lerner et al., 2012 (96) [pathway]		Non-thyroid cells, ERS–NLRP3 pathway [non-thyroid]; HT patient tissue + HT/EAT models, pyroptosis [HT/EAT]	Causal (non-thyroid pathway); Correlative (HT tissue, Guo); Causal (HT/EAT, USP1 inhibitor)	IRE1α-TXNIP-NLRP3 and PERK-NF-κB-NLRP3 established in general biology; NLRP3 pyroptosis confirmed in HT tissue with autoantibody correlation; USP1 inhibitor improved HT pathology	IRE1α-TXNIP-NLRP3 activation not measured in HT thyroid tissue
Ferroptosis (Section 4.4).						
ERS-driven ferroptosis	Sacristán-Gómez et al., 2026 (97); Mou et al., 2025 (98) [HT]; Dixon et al., 2014 (99) [pathway]; Chen et al., 2025 (100) [pathway]		Non-thyroid cells, ERS–ferroptosis pathway [non-thyroid]; HT patient tissue, EAT rat model [HT/EAT]	Causal (non-thyroid pathway); Correlative (HT tissue); Causal (EAT model)	PERK-ATF4-CHAC1 depletes GSH and inactivates GPX4 (general biology); GPX4 decreased and 4-HNE elevated in HT tissue; ursolic acid restored GPX4/SLC7A11 and Th17/Treg balance in EAT rats	PERK-ATF4-CHAC1 activation not measured in HT thyroid tissue

Evidence tier ranks proximity to HT pathology (“>“ denotes decreasing proximity): [HT/EAT] = HT/EAT tissue or model (findings obtained in HT patient tissue or in experimental autoimmune thyroiditis/HT animal models) > [thyroid-cell] = Thyroid-cell system (thyroid-derived cells or thyroid-specific models, typically under pharmacologically induced ERS) > [non-thyroid] = Non-thyroid system (mechanisms extrapolated from non-thyroid cells or models). Evidence type records how a finding was established: Causal — demonstrated by perturbation (knockdown, knockout, pharmacological inhibition/rescue) or temporal/ordering experiments; Correlative — a measured association without perturbation (e.g., marker co-variation, pathway enrichment, genetic association); Descriptive — qualitative observation only (e.g., ultrastructural morphology), without quantified association or perturbation. In the System & evidence tier column, each system carries its proximity tier in brackets; rows spanning multiple systems list them separated by “;”. In the Evidence type column, multiple types are separated by “;”, each annotated in parentheses with the system or scope from which that inference derives, so that the directness of every causal, correlative, or descriptive claim can be traced to its tier within a single line. AITD, autoimmune thyroid disease; EAT, experimental autoimmune thyroiditis; ERAD, ER-associated degradation; ERS, endoplasmic reticulum stress; GD, Graves’ disease; IHC, immunohistochemistry; TEM, transmission electron microscopy; UPR, unfolded protein response.

### 6.2 Reasonably extrapolated evidence and its logical boundaries

ERS-mediated thyrocyte injury modes. The molecular mechanisms of four injury modes—apoptosis, Tg processing defects, thyroid-specific gene suppression, and dedifferentiation/EMT—are established almost entirely in thyroid cell lines under pharmacological ERS inducers (28–32); the differentiated thyroid cell lines used and the HT-consistent direction of functional loss support extrapolation, and a chronic misfolded-Tg model partially offsets the acute-induction limitation (31). Still, acute global UPR may differ from the chronic, partial ERS of HT *in vivo*, and no study has quantified these injury modes in HT patient tissue, so their actual contribution cannot be determined.

UPR regulation of immune cells. With the exception of macrophage IRE1α, UPR evidence for T cells and dendritic cells is non-HT, and the plasma cell lineage has only HT scRNA-seq correlation without branch resolution or functional validation (**Table 1**). It should be emphasized that the extrapolated nature of this evidence does not equate to low quality: conditional knockout experiments from non-HT systems may surpass correlative observations in HT patient tissue in causal validation strength. The core uncertainty is whether the HT microenvironment, with sustained Tg autoantigen stimulation, abnormal iodine metabolism, and a distinct cytokine profile, alters the UPR patterns established in other autoimmune or tumor models.

ERS and programmed cell death. Pyroptosis and ferroptosis occur in HT thyroid tissue (60, 95, 97, 98), and the pathways by which ERS drives them are established in non-HT models (96, 99, 100). The break in the chain is precise: whether IRE1α activates NLRP3 via TXNIP, and whether ATF4 drives ferroptosis via CHAC1-mediated GSH depletion, has not been examined in HT tissue.

The respective boundaries of the above three categories of extrapolation collectively point to a more fundamental question: where ERS sits within the causal chain of HT. This review organizes ERS as a node connecting thyrocyte injury and immune dysregulation—a narrative that implicitly assumes a relatively upstream position for ERS; however, the current evidence is insufficient to establish this causal direction. The heritability of HT is approximately 0.64, with risk loci concentrated primarily in HLA and other immune-regulatory loci (5); single-cell and histological studies also indicate that follicular destruction is driven by infiltrating immune cells and stromal components, a process that is mechanistically capable of proceeding without ERS as an intermediary (6). These lines of evidence do not exclude the participation of ERS but suggest that immune-intrinsic mechanisms may occupy a more upstream position, and that thyrocyte ERS may be a secondary consequence of the inflammatory microenvironment rather than its initiating cause. The two are not mutually exclusive: even if HT is initiated by immune mechanisms, ERS may still function as an amplification step contributing to the maintenance and progression of established lesions. The critical evidence capable of distinguishing between these two possibilities—the temporal relationship between ERS and immune infiltration in early human HT lesions, and the causal effect of thyroid-specific ERS intervention on autoimmune progression—is currently entirely absent. Accordingly, the evidence integrated in this review aligns more closely with the amplification and maintenance level of injury, rather than the tolerance-breakdown initiation level; ERS should be positioned as a mechanistically plausible candidate amplification node that awaits causal validation.

### 6.3 Experimental validation pathways for the positive-feedback perspective

Following the above evidence synthesis and stratification, a testable positive-feedback perspective can be integrated (**Figure 1**): it is not an established pathway but an analytical framework for organizing existing evidence and exposing its gaps. This perspective comprises six key steps (1): ERS activation in thyrocytes (2); ERS-mediated thyrocyte injury releasing DAMPs and conformationally altered autoantigens (3); DAMPs/antigens activating infiltrating immune cells (4); intra-immune-cell UPR regulating their differentiation and effector functions (5); pro-inflammatory cytokines such as TNF-α and IFN-γ secreted by immune cells retrograde-amplifying thyrocyte ERS; and (6) establishment and self-maintenance of the positive-feedback loop. Among these, Step (1) and the macrophage IRE1α pathway within Step (4) have received direct HT/EAT validation; all remaining steps are inferential. The one partial exception is flagged in **Figure 1**: thyrocyte apoptosis/necroptosis has been directly observed in HT tissue (12), but its dependence on ERS has been demonstrated only in thyroid cell lines.

Testing this positive-feedback perspective requires filling evidence gaps at three levels. First, direct detection of immune-cell UPR: beyond macrophages, directly examining UPR pathway activation in HT thyroid-infiltrating T cells, plasma cells, and dendritic cells represents the highest-priority experimental direction. Second, causal validation (Steps 4–5): even if UPR activation is detected in the above immune cells, loss-of-function experiments demonstrating that UPR inactivation attenuates HT/EAT immunopathology are required to exclude the possibility that UPR activation is merely an accompanying phenomenon. Third, experimental closure of the loop (Steps 5–6): it is necessary to demonstrate that inflammatory factors released by activated immune cells can retrograde-induce UPR upregulation in thyrocytes, thereby confirming the existence of transcellular positive feedback. These strategies are constrained by existing models: EAT relies on active immunization and differs from spontaneous human HT, and HT surgical specimens, mostly from nodule or goiter cases, poorly represent early-stage tissue.

Simultaneously, articulating these six steps as an integrative perspective requires explicitly stating the conditions under which it can be falsified. If ERS in HT tissue is simultaneously regarded as the cause of injury, the mediator of immune regulation, and the downstream consequence of inflammation, then ERS detected at any stage, in any cell type, could be interpreted as supporting evidence—a proposition that would lack scientific meaning. Given that ERS is a ubiquitous cellular stress response, the complete absence of ERS activation in HT tissue is not a realistic falsification condition; the falsifiability of this perspective must be grounded in tests of directionality, specificity, and causality. Accordingly, the following observations would falsify or substantially weaken the positive-feedback perspective. First: if thyrocyte ERS markers are absent in early HT lesions where immune infiltration has not yet been established, or if temporal studies reveal that immune cell activation precedes thyrocyte ERS, then the positioning of ERS as an upstream initiating event would be invalidated. Second: Section 3.2.5 has already noted that DAMPs detected in HT tissue cannot be attributed to ERS; if it is further demonstrated that the immune-activating signals released by ERS-mediated thyrocyte death are qualitatively indistinguishable from those released by apoptosis, necrosis, pyroptosis, and other death modalities, then the claim of ERS as a specific pathogenic node distinct from cell death per se would collapse. Third: as the inverse result of the second validation pathway above, if specific inactivation of UPR in immune cells (e.g., macrophage IRE1α conditional knockout) fails to attenuate EAT immunopathology, then immune-cell UPR should be judged an accompanying phenomenon rather than an effector mechanism. Fourth: if pro-inflammatory cytokines such as TNF-α and IFN-γ cannot retrograde-induce UPR upregulation in thyrocytes, then transcellular positive feedback is not supported.

### 6.4 Limitations of this review

This review has four methodological limitations. First, the direct HT/EAT evidence base is thin and unreplicated. As noted in Section 6.1, direct evidence is limited to Step 1 and the macrophage IRE1α pathway. Step 1 relies on one localizing study plus whole-thyroid corroboration, whereas Step 4 rests on a single study. Moreover, the two-patient single-cell atlas cannot capture HT heterogeneity. Second, as a narrative review, it performed no formal risk-of-bias assessment. With a small ERS-HT evidence base concentrated in a few research groups, selection and publication bias may inflate the apparent consistency. Third, the evidence stratification reflects disease directness, not study quality: directness and causal strength are independent dimensions, so a correlative HT-tissue observation outranks a non-HT conditional knockout in directness, while the knockout may be stronger in inference. Fourth, only English-language literature was included; given active autoimmune-thyroiditis research in the traditional Chinese medicine field, relevant ERS data in Chinese-language journals may have been missed, affecting completeness.

## 7 Conclusion

This review has synthesized dispersed ERS evidence from thyroid cell biology and immunology, stratified by its proximity to HT/EAT pathology. On this basis, a testable positive-feedback perspective is proposed: thyrocyte ERS injury and immune effector amplification may mutually sustain one another. This perspective is an analytical framework for organizing evidence and exposing gaps, rather than an established pathway. Among its six steps, only thyrocyte ERS activation and the macrophage IRE1α pathway have received preliminary direct validation; the remaining steps lack HT-specific evidence.
